# Textile-Based Stretchable Microstrip Antenna with Intrinsic Strain Sensing

**DOI:** 10.1021/acsaelm.1c00179

**Published:** 2021-05-03

**Authors:** Fatemeh Nikbakhtnasrabadi, Hatem El Matbouly, Markellos Ntagios, Ravinder Dahiya

**Affiliations:** Bendable Electronics and Sensing Technologies (BEST) Group, University of Glasgow, Glasgow G12 8QQ, U.K.

**Keywords:** e-textile, antenna sensor, strain sensor, wearable electronics, multifunctional antenna

## Abstract

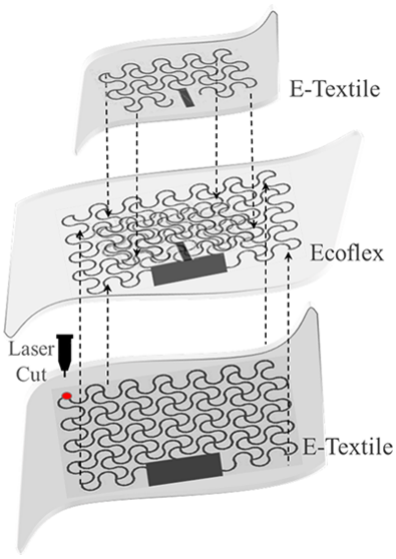

This paper presents a textile-based stretchable microstrip patch antenna with intrinsic strain for e-textiles with seamlessly integrated multifunctional devices. Several microstrip antennas have been developed with the patch alone (stretchable up to 40%) or both the patch and the ground plane (stretchable up to 100%) meshed by using rectangular serpentine units. The changes in the resonant frequency of the meshed antennas, as a result of stretching, have been exploited to demonstrate the intrinsic uniaxial strain sensing. The obtained results indicate that resonant frequency decreases linearly with increasing applied strain, suggesting that the designed antennas can also be used as strain sensors with stretchability up to 100% and a sensitivity of 0.25. The results were validated through full-wave electromagnetic simulations and a two-dimensional digital image correlation (DIC) technique to model the antenna deformations during the tensile tests. In terms of stretchability, the meshed textile patch antenna on a solid ground plane showed more than a 2-fold improvement compared to a meshed gold patch antenna, showing a linear frequency shift. As potential applications, we demonstrate the use of a highly deformable fully meshed textile antenna as a strain sensor capable of measuring joint angles of a human limb. To do that, a microwave readout circuit based on RF to DC rectifier was realized. The rectifier obtained a peak conversion efficiency of 71% at 10 dBm input power overload resistor of 3 kΩ.

## I Introduction

The increasing use of wearable systems for wellness and health monitoring applications has directed the attention of researchers toward wireless sensor networks (WSNs) and body sensor networks (BSNs) for real-time measurement of physiological parameters such as heart rate, body temperature, strain, body motions, and so on.^1−5^ Some of these wireless sensors also find application in humanoid robotics, prosthetics, and human–machine interactions, particularly to address the issue of wiring complexity.^6−8^ The flexibility and conformability of more and more functional devices have been the trend in recent years to meet the key requirements of these applications. This has been generally achieved through rigid tiny sensors (nodes) integrated over the polymeric substrates and connected via wavy metallic interconnects. However, the wiring required to acquire the data is prone to damage, for example, during bending in wearable systems, and may lead to interferences between parallel signals as well as time delays.^9^ Likewise, the increasing number of devices also raises integration challenges, in addition to traditional challenges such as higher cost and power requirements. Considering these challenges, a system with a similar level of functionality (as with a large number of nodes and sensors) and yet less complexity is much needed. In this regard, multifunctionality (e.g., communication as well as sensing) with commonly used devices such as antennas could be an interesting way forward. As an example, the flexible and stretchable antennas in wearable systems always experience some sort of deformation, and as a result, the response changes as well. The change in the response (e.g., shift in resonant frequency^10^) carries the signature of deformation, which reflects the potential route for using the antenna as a strain sensor and a way to decrease the number of components without sacrificing the functionality or increasing the integration challenges. The work presented in this paper demonstrates such a multifunctional antenna realized with stretchable conductive textile.

**Figure 1:**
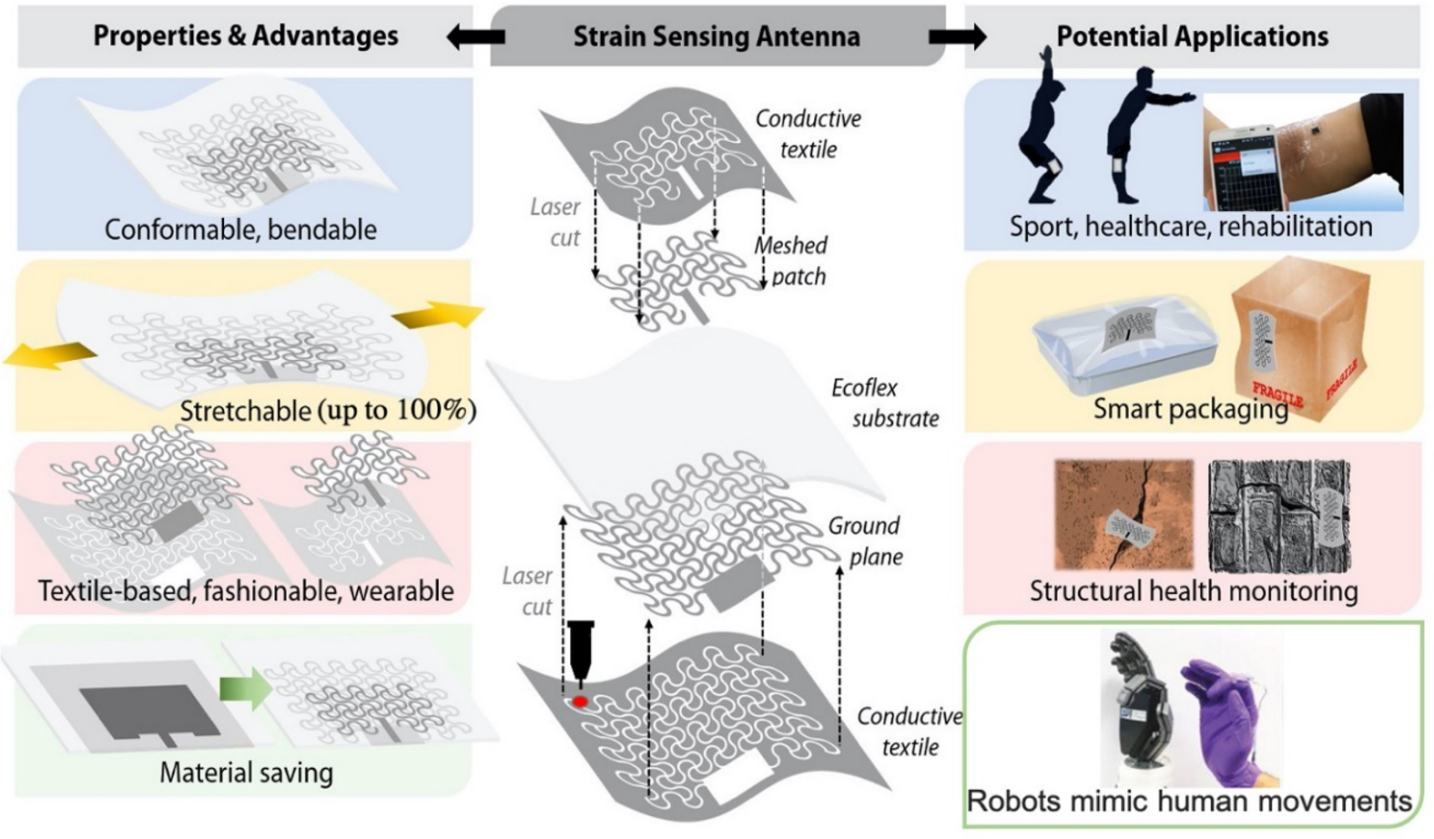
Overview of the textile-based microstrip antenna with intrinsic strain sensing with major advantages and potential applications. The image on top right corner reproduced from ref (16). Copyright 2018 Elsevier. The image on bottom left corner reproduced with permission from ref (51). Copyright 2020 Wiley-VCH.

The conceptual overview of the developed textile-based microstrip antenna is shown in Figure 1. The applications shown in Figure 1 experience stretching, and multiple use antennas can be advantageous as a reduced number of electronic components will be required. For example, with stretchable antenna-based strain sensor on joints of a robotic hand, it is possible to detect the joint angles and use this information to control the hand movements. Likewise, the stretching of antenna with inflated food package (due to food spoilage) will reflect the state of food inside the package. Furthermore, the stretchability of textile antenna can improve wearability, if the presented devices are used as part of wearables. The textile-based stretchable microstrip antennas demonstrate a linear resonant frequency shift when the antennas are elongated due to external uniaxial strain, and based on this the feasibility of “antenna as a strain sensor” has been explored. Therefore, for potential application, the RF readout circuit is the one which is going to be used. The DC output represents the amount of stretch applied to the antenna and can be used to actuate other devices. The designed textile-based antenna sensors show an average linear sensitivity of 0.25 to external uniaxial strain up to 100% uniaxial strain. For demonstration purposes, we have embedded the textile in biocompatible elastomers. However, a blend of polymer and conventional textile (just as waterproof clothes) could be explored, too.

The general method to convert the conventional electrodes to stretchable ones consists of patterning them with a repeated unit cell. Generally, the stiffness of metals could be managed by using this approach to fabricate highly stretchable 3D structures.^11^ Kirigami-like structures,^12−14^ twisted helical springs,^15^ and serpentine shapes^16−18^ are some of the conventional methods to develop large-area electronics with duplicated unit cells, either to enhance stretchability or minimize the size.^19^ These methods have been rarely reported as an active or passive microwave device such as an antenna.

This paper is organized as follows: Section II presents the state of the art related to stretchable antennas and the general strategy employed in this work to realize stretchable textile-based microstrip antennas. Fabrication methods and materials used to develop the stretchable antennas are described in section III. The methodology to characterize the designed antennas is also described in section III. Simulated and experimental results are explained in section IV, and last, the key outcomes are summarized in section V.

## II State of the Art

Various types of stretchable antennas have been reported in recent years to match the requirements of wearable system applications.^20−22^ The choice of the antenna structure in this application depends on the parameters such as available space, operating frequency range, and application. In most of the work reported thus far, the sensor and antenna are separated devices. There are only a few works where these functionalities have been integrated and used as a single device.^23,24^ As an example, a dipole antenna with arms designed in a meander line shape has been reported.^25^ In many of the works reported for wearable applications, the challenge is to minimize the dissipation of electromagnetic radiation by human tissues. In this regard, the microstrip patch antennas with a ground plane have advantages over the dipole-based antennas. For this reason, the microstrip patch antenna has been used in this work to demonstrate the concept of the multiuse antenna.

**Figure 2:**
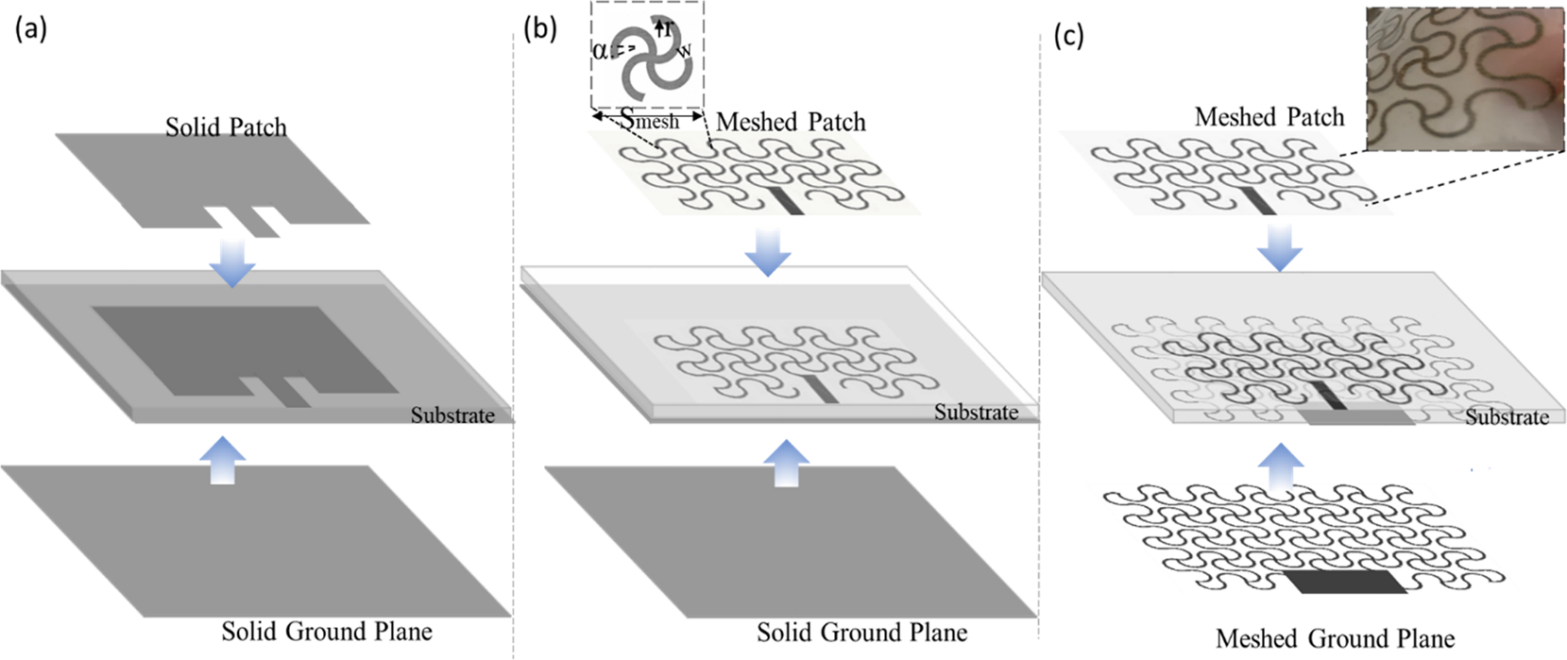
Schematic view of the three versions of the designed textile microstrip antennas. In all cases, the substrate is Ecoflex with thickness of 2 mm, and the overall sizes of the patches and ground planes were kept equal. (a) Microstrip antenna made of solid metal-plated conductive textile. (b) Improving stretchability of microstrip antenna by converting the solid patch to a meshed structure. The ground plane is kept as solid metal-plated textile. (c) Conversion of both the solid patch and the ground plane with meshed structures. The inset image demonstrates that meshed structures are embedded in Ecoflex because Ecoflex was partially cured.

A microstrip patch antenna consists of a radiating metallic patch layer placed on one side of a nonconductive substrate and a ground plane on the other side (Figure 2a). Ideally, an infinite ground plane has a significant role to eliminate back-radiation, but finite ground planes are widely used in real applications.^26^ These low-profile antennas can conform to various surfaces and are therefore suitable for wearable applications. They are also versatile in terms of resonant frequency, radiation pattern, directivity, and polarization. Importantly, they could also be used as strain sensors. To this end, a common method in the literature is based on tracking the shift in resonant frequency to gauge the occurred deformation.^27^ As an example, applying tensile strain along the feeding direction of a stretchable microstrip antenna can cause a downshift in the resonant frequency. However, the maximum applied strain was 15% due to the limited stretchability of the antenna.^18^ To increase the range of applied strain, different geometries can be implemented. Table 1 shows a comparison of stretchable microstrip patch antennas that can be found in the literature in terms of stretchability and sensitivity to strain. As can be noted from this table, the antennas presented in this paper can stretch much longer than previously reported antennas while maintaining a good average sensitivity.

**Table 1:** Performance Comparison of Our Proposed Textile Stretchable Microstrip Antennas with Other Stretchable Antennas Reported in the Literature

stretchable unit cell	conductive material	fabrication method	stretchability (%)	sensitivity	ref
hierarchical triangular lattice	copper foil	cutting with programmable desktop cutter machine	15	0.82	(19)
N/A	AgNWs	drop casting the AgNWs on PDMS	15	0.245	(22)
rectangular mesh	liquid metal alloy	injecting liquid metal into microchannels	20	0.552 at 15% strain	(28)
serpentine rectangular meshed patch over solid ground plane	conductive textile	cutting with laser cutter	40	0.20 (avg)	this work (patch is meshed)
serpentine rectangular meshed in both patch and ground planes	conductive textile	cutting with laser cutter	100	0.25 (avg)	this work (both patch and ground plane are meshed)

To realize the antennas, a wide variety of conductive materials have been explored, including Ag-PDMS composite^28^ and metal nanowires.^29,30^ More recently, the liquid metal alloys also such as eutectic gallium–indium (EGaIn) and galinstan have been explored as they are highly deformable and can be processed at room temperature.^31^ In addition, they could be patterned by lithographic methods^32,33^ and injection of liquid metal into microfluidic channels^34^ to form a flexible and stretchable antenna. Despite the recent developments in stretchable and flexible conductive materials, poor conductivity is a bottleneck to deploy these materials in radio-frequency (RF) wireless technologies. To address the challenges, CNTs-composite-based deformable antennas have been reported.^18,35^ In a different approach, the use of conductive textiles composed of a polymer-based fabric coated with conductive materials such as copper and silver could address the above-mentioned issues. One of the advantages of using e-textiles with high conductivity is the reduced cost of integrating an antenna into garments. Moreover, our proposed fabrication method has the advantage of having high stretchability which can improve conformability to the human body, while exhibiting the sensitivity to strain in the same range as published works (as summarized in Table 1). This differentiating feature of our work can also open the doors for more different implementations where more stretching is required.

Emerging fabrication techniques also make it feasible to pattern conductive textiles into an antenna shape. For example, antennas with compound geometries can be fabricated by using either computerized sewing machines or automated laser cutters. The thread size, woven pattern, and stitch density have an impact on the appearance and performance of the antenna. By by use of these fabrication tools, it is possible to develop textile antennas with significantly enhanced conformability compared to the traditional rigid antennas made from conventional conductive materials such as silver and copper sheets.

## III Design and Fabrication

### Antenna Design

The schematic view of the three-microstrip antenna design used in this work is shown in Figure 2. Similar designs, converting a solid antenna structure into a meshed layout for RF applications, have been reported in the past.^36^ The stretchability of meshed structure increases with the larger serpentine arc angle. Based on the study that has been done in ref (45), there is a trade-off between mechanical stretchability and EM performance of meshed antennas. It was shown that the attenuation constant (α), which is an indicator of propagation loss, is minimum when the serpentine arc angle is 220°. Considering the limitation of chosen fabrication method (cutting with laser), the length of each unit, which indicates the density of meshes, is chosen as 15 mm, which is in the subwavelength range, about λ/15 (λ is free space wavelength). The effect of line width is negligible^50^ on the resonance frequency; however, to keep the radiation loss minimum as possible the minimal value of 1 mm is chosen as line width, which is the smallest feature of the textile that can be cut by using the laser cutter. Figure 2 illustrates the general strategy that was followed to convert the solid antenna to a fully stretchable structure. First, a conventional textile microstrip patch antenna was designed (Figure 2a). Based on that design, the patch layer was then patterned in a meshed layout to improve the stretchability of the antenna (Figure 2b).

Afterward, the ground plane was also converted to a meshed layout, thus achieving up to 100% stretchability (Figure 2c). In the following sections, the electromagnetic performance of these three textile antennas is compared in terms of resonant frequency and radiation pattern.

The dimensions of the antenna are the key factors that define its resonant frequency. To design a rectangular patch antenna, the length and the width of the rectangular patch were calculated based on the transmission line model. The four most popular feeding techniques to feed microstrip antennas, i.e., to drive power to the antenna, are coaxial probe, microstrip line, aperture coupling, and proximity coupling. To realize a planar structure and eliminate the need for additional matching elements, the proposed antenna was fed by using an inset feed microstrip line. Generally, the input impedance of the patch in the edge is almost 300 Ω. Because most microwave sources are manufactured with a characteristic impedance of 50 Ω, an inset feed line was used to match the impedance of the source and the patch. Inset point and size of slots were obtained through optimization in High-Frequency Structure Simulator (HFSS) software (ANSYS Electromagnetics Suite 19.0, USA). According to the desired dimensions of the antenna (45 mm × 26 mm), the main resonant frequency was found at 4 GHz for the case of the solid textile patch antenna. According to Ofcom,^37^ the 3.8–4.2 GHz spectrum band could enable the use of 5G technology for private industrial networks. The dimensions of the ground plane were kept 6*h* + *L* and 6*h* + *W* as suggested in the literature.^38^ The ground plane was made from the same conductive textile with dimensions of 57 mm × 38 mm. The threshold for reflection coefficient was considered −20 log|*S*_11_|< −10 dB, meaning that 90% of incoming power in the desired resonant frequency is radiating, where dielectric and ohmic losses are negligible. The slot width adjacent to the feedline was 3 mm, which was inset into the rectangular patch for 9 mm.

### Materials

In contrast to the conventional rigid antennas, textile-based antennas are flexible and conformable and fabricated by using a wider variety of conductive fabrics.^39,40^ In this work, silver-plated knitted textile (MedTex, UK) was used to fabricate the radiating patch and ground plane of microstrip antennas, while Ecoflex was sandwiched in between the two layers as the substrate. The employed textile sheet was made of 94% nylon and 6% elastomer plated with 99% pure silver. The total thickness of the textile sheet was 0.55 ± 10% mm with an average conductivity of 1.14 × 10^3^ S/m. The average thickness of plated silver was ∼0.015 mm.^41^ This textile sheet was selected due to its stretchability without significant change in its resistance. All the mentioned values were assigned as the conductive textile material properties in the HFSS model.

In wearable applications where the deformability of the antennas plays an important role, rigid substrates such as FR4 can be replaced by polymer materials such as PDMS, Ecoflex, Solaris, and so on. Among these, transparent and hyperelastic polymers such as Ecoflex are preferred as they show higher viscosity, and hence the interfacial bonding between the substrate and the textile is very strong. Moreover, Ecoflex is an ultrasoft polymer material with a Young’s modulus of 125 kPa, indicating good conformation with human skin in the case that the device is placed directly on human skin for monitoring applications.^42^ In this work, 2 mm thick Ecoflex 00-30 (Smooth-On Inc., USA) was used as a substrate for all samples. The polymer was supplied in two parts, i.e., base and curing agent (A and B). In the simulation model, a dielectric constant of 2.8 and loss tangent of 0.02 were assigned to Ecoflex considering the mixing ratio 1A:1B.^43^

### Textile-Based Antenna Fabrication

Three versions of textile-based microstrip antennas, as shown in Figure 2, were fabricated by using the laser cutter. To fabricate the first version (Figure 2a), which is a conventional microstrip antenna made from the stretchable conductive textile, the patch and ground plane outlines were cut by using the laser-based cutting machine according to the designed dimensions (see section III). For the Ecoflex 00-30 substrate, parts A and B were mixed well with a 1:1 ratio. The mixture was then degassed in a desiccator for 15 min to remove bubbles. Afterward, the mixture was poured into a rectangular mold made of poly(lactic acid) (PLA) with 2.5 mm depth. Once Ecoflex was partially cured, the patch was placed on top. Because the mixture was not fully cured at this point, the patch was slightly embedded within the substrate, thus ensuring good adhesion between them and sealing the textile. The porous nature of the fabric allows the creation of polymer–polymer bonding that provides good adhesion. Afterward, the cured Ecoflex was peeled off from the mold, and the ground plane was bonded to the substrate underneath by using a very thin layer of fresh Ecoflex. At this point, it was observed that the strong adhesion limited the stretchability of the textile in a way that the antenna was not stretchable anymore, despite the stretchy nature of the used textile. Eventually, an edge-mount Subminiature Version A (SMA), 50 Ω, RF connector was attached to the antenna feed line. Because the materials used to fabricate the antenna were sensitive to high temperatures, the SMA connector was attached by using room-temperature curable silver conductive adhesive epoxy (MG Chemical, UK) instead of soldering.

**Figure 3:**
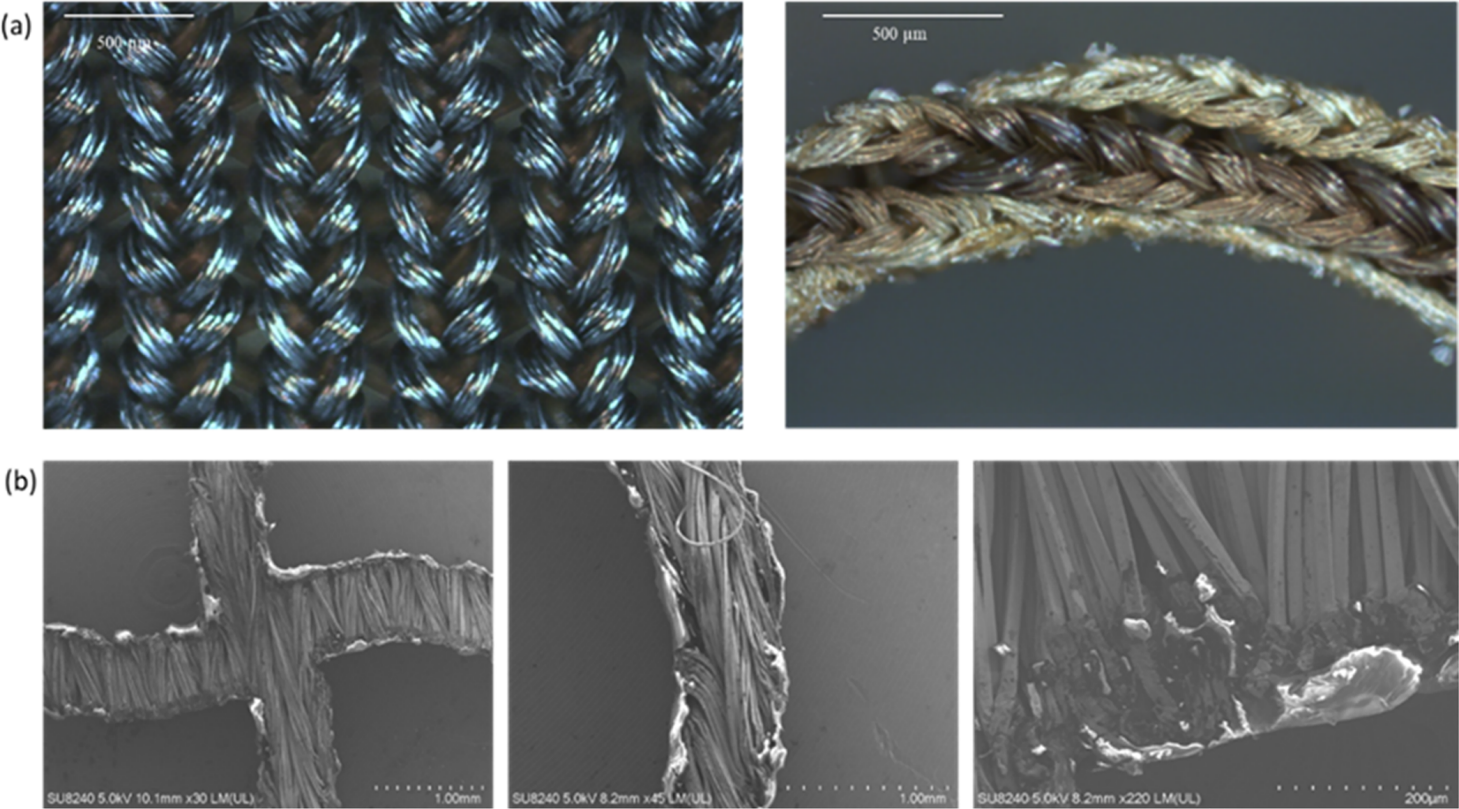
(a) Optical images of silver-plated knitted textile (left) and laser cut textile (right). (b) SEM image of laser cut textile of a random joint point and magnification on cut edges where nylon/elastomer was melted due to laser operation.

The first step to overcome the lack of stretchability in the first version of the textile antenna was to pattern the patch in a rectangular serpentine structure. The solid patch was converted to a serpentine-shape layout as shown in Figure 2b. The antenna stretchability is highly dependent on the morphologic parameters of the serpentine pattern, such as the radius of curvature, arc angle, and tracks width. The arc angle was set to 220° according to the previous study,^44^ which results in less absorption loss while maintaining mechanical robustness. Figure 2b inset shows the serpentine-unit pattern, which was periodically implemented throughout the entire structure. The internal radius and the thickness of each arc unit were 2.25 mm and 1 mm, respectively. These values were chosen considering the resolution limitations of the laser cutter. The material database of a laser cutter in terms of power (optimum value = 20%), speed (optimum value = 3), and pulse per inch (optimum value of PPI = 1000 Hz) were tuned to prevent burning marks in the edges of the design. The connection track between the feedline and the meshed patch was slightly wider (i.e., 1.5 mm) than the other tracks to avoid failure upon stretching. The pattern was first designed in AutoCAD and then imported to Corel Draw for the cutting process with the automated laser cutter. Figure 3a shows a microscope view of the silver-plated textile before and after cutting. Although some outlines with burn marks can be observed, the resistance did not change significantly. The resistance of the serpentine unit (end-to-end) was 1.8 ± 0.17 Ω. Figure 3b shows scanning electron microscopy (SEM) images of random joint points of the fabricated pattern at different magnifications. White marks show that the polymer was slightly melted in the edges by the laser. Lastly, the meshed patch and solid ground plane were bonded to the Ecoflex substrate, and an SMA connector was attached as described earlier.

To achieve a fully meshed microstrip antenna with higher stretchability, the solid ground plane was also converted to a meshed pattern (Figure 2c). The effect of replacing the solid textile with a meshed pattern on the resonant frequency and radiation pattern will be explained in the next section. In this stage, the textile was laser cut by using the same method, as described before to pattern the patch, to fabricate meshed ground plane. It is worth mentioning that the 1 × 2 mm^2^ portion in the midpoint was kept solid to provide durable support to connect SMA to the ground plane. Likewise, as previously described, both meshed patch and ground planes were attached to the substrate and an SMA connector.

### Gold Meshed-Patch Antenna Fabrication

**Figure 4:**
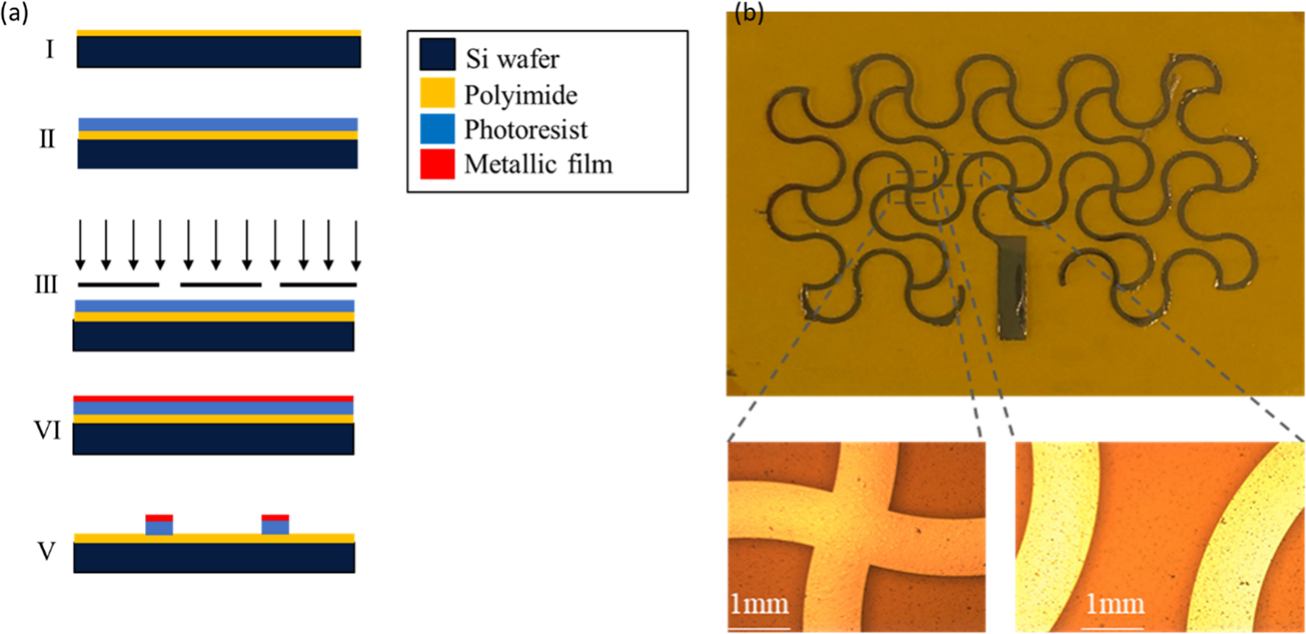
(a) Schematic representation of the fabrication steps for realization of gold patch on polyimide. (b) Optical microscope image of random parts of the gold pattern.

To compare the performance of our textile-based microstrip antenna (i.e., meshed patch with a solid ground) with its metal-based counterpart,^18,45^ a gold (Au) meshed antenna with the same dimensions was fabricated by using photolithography steps.^46^ To this end, a polyimide (PI) layer (DuPont Inc., Wilmington, DE) was spun on a carrier wafer. Then it was cured for 3 h at 200 °C in ambient nitrogen to form a sheet of 25 μm thickness. Later, a positive photoresist (S1818) was spun (4000 rpm for 30 s) on it and baked on a hot plate at 115 °C for 3 min. The pattern was defined by using a high-resolution optical mask aligner (SUSS MicroTec., Germany) with UV exposure for 6 s. Then a MF-319 developer was used for 2.5 min to form the pattern. Metal evaporation of Ti(10 nm)/Au(100 nm) formed the layer of metal deposited on the PI substrate. Subsequently, a lift-off stage in an ultrasonic acetone bath was performed. Figure 4a depicts the fabrication process, while Figure 4b shows the sample after being rinsed gently in isopropyl alcohol (IPA) to remove any gold residues. Optical microscope images (Figure 4b) confirm that gold tracks are intact without any crack, and the adhesion between the gold layer and the PI film is robust. Eventually, the unpatterned part of the PI film was removed by using the laser cutter, and the patch was attached to the Ecoflex substrate following the same procedure described for the textile-based microstrip antennas. Commercial single-side copper clad board (Kitronik, UK) with a thickness of 1 mm was used as a free-stand ground plane beneath the sample.

### Experimental Characterization Details

**Figure 5:**
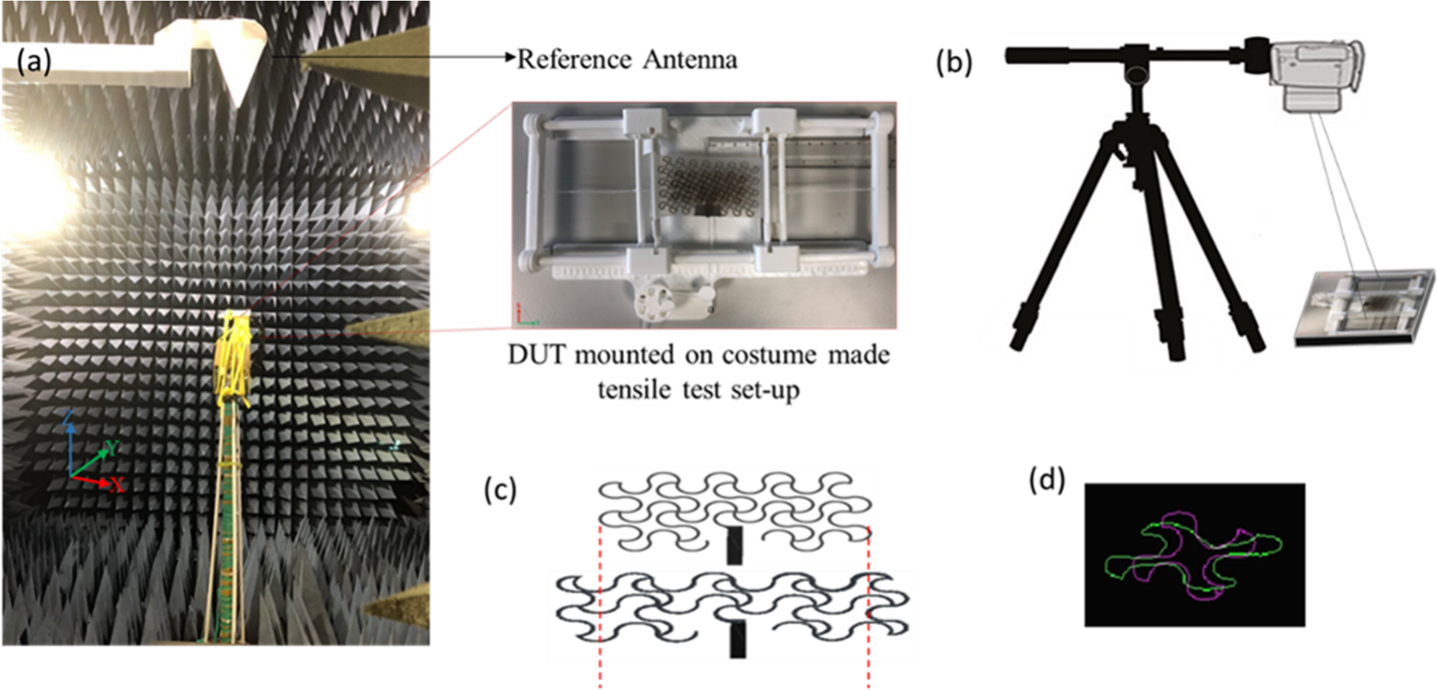
(a) Experimental setup for radiation pattern measurements inside the anechoic chamber (left). Broadband (0.5 MHz–18 GHz) dual polarized log periodic antenna was used as reference. The inset image on the right shows the fully meshed microstrip antenna mounted on the custom-made setup in zero strain condition. (b) 2D DIC setup with horizontal tripod arm. The optical axis of the camera is aligned perpendicular to the sample surface to avoid errors in the image correlation. (c) Representative serpentine unit cell of the patch. Purple contour shows an undeformed shape while green one shows the corresponding contour for 40% of strain. (d) CAD schematic representing the surface morphology of the patch in 0% and 40% of strain.

To characterize the fabricated prototypes, a custom-made setup was used to evaluate their performance over uniaxial stretching (Figure 5a). The holder is 3D-printed by using an Ultimaker S5 and poly(lactic acid) (PLA) filament (RS Components, UK). Two parallel grooved grippers were used to hold the sample in place, and uniform uniaxial tension was applied to the samples by pulling the grippers with the help of a light rope and a pulley system. Grade markers were printed on the setup to measure the elongation while applying uniaxial strain. For RF characterization, the scattering parameters of the antennas were collected by using a vector network analyzer (VNA) (E83628, Agilent Technologies, USA) to observe the change of resonant frequency with the applied strain. Radiation pattern measurements were conducted inside an anechoic chamber (Figure 5a).

To validate the experimental results, all the proposed structures were simulated in HFSS. The stretching procedure was recorded by using a high-resolution camera and digital image correlation (DIC) technique to monitor the deformation of the meshed layouts, as described below. Processed images acquired at a specific stage of stretching were imported into HFSS to evaluate their electromagnetic (EM) performance. The mechanical deformation of serpentine mesh geometries upon the uniaxial strain has been investigated in past by using the finite element method (FEM) through ABAQUS with some simplifications (e.g., considering uniformity in strain to the entire model and out of plane deformation as in-plane deformations) applied due to the excessive number of nodes and elements.^18,36^ It is worth mentioning that the conductive material in previous studies was a conventional copper sheet. In our study, commercial conductive knitted textile was used, in which actual material properties such as Young’s modulus, Poisson’s ratio, and the coefficient of friction are unavailable. Moreover, the nonplanar nature of textiles and the effect of mechanical distortion on the inhomogeneous displacement of the textile yarns make FEM an unreliable technique for our textile antennas. As an alternative, a two-dimensional DIC technique was used to evaluate the applied strain by using the contactless full-field optical technique over the sample area during uniaxial tensile tests. DIC provides an accurate solution to investigate material deformation and crack propagation in real-world applications.^47^ The basic principle of this technique is to monitor displacements by analysis of a digital image series. This is done by taking successive images of the objects over time under various uniaxial strain conditions and then analyzing them through specific correlation-based algorithms.^48^ To apply this technique, the camera was positioned vertically and parallel to the flat surface of the sample to avoid out-of-plane image correlation errors.^49^ Because only one camera is used in this technique, it cannot record out-of-plane displacement as well; however, in our experiment there is no out-of-plane displacement. The recorded video was split into a series of digital images for postprocessing in MATLAB to obtain a full-field deformation map at different levels of elongation. The effect of uniaxial strain was indirectly determined considering differentiation of the measured displacements. Afterward, the pixel displacements were converted to actual displacements to model the deformed patch in AutoCAD and then imported to HFSS to investigate the radiation patterns in different levels of applied strain. It was observed that by increasing the tensile strain, the vertical arcs became shorter and horizontal arcs became wider. However, serpentine units were deformed perpendicular to the feedline direction rather than in the direction of the current flow. Figure 5b shows a schematic of the DIC setup for in-plane displacement measurement. As a proof of concept, the pristine and deformed shape of the unit cell and patch, obtained by using the DIC technique, are presented in Figures 5c and 5d for strains of 0% and 40%.

## IV Results and Discussion

**Figure 6:**
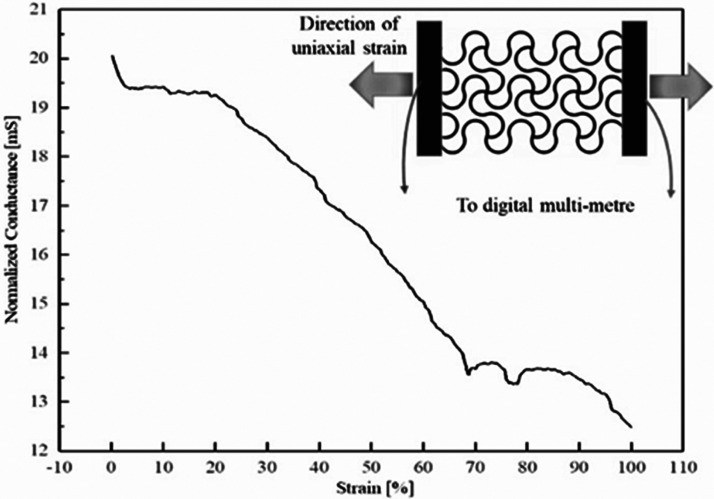
Change in conductance of textile meshed patch at different values of uniaxial tensile deformation. The inset image shows a schematic of the electromechanical test setup.

In the first place, the custom-made tensile setup was used to study the change in the electrical properties of the textile (i.e., resistance) upon uniaxial stretching. Figure S1 demonstrates the setup that was used to evaluate the conductance of the patch in terms of change in DC voltage as a function of uniaxial strain. Figure 6 shows that the end-to-end conductance of the patch halved when strain increased gradually up to 100%. The average conductivity of the patch was 0.89 × 10^3^ and 0.58 × 10^3^ S/m under 0% and 30% strain, respectively. The corresponding value of conductivity is still good enough to be used as a radiating structure in antennas.^45^ Furthermore, to evaluate fatigue cycling stability of the meshed patch, a 100-cycle test was performed, and it was observed that it retained its original value with standard deviation of 5.8% under 30% strain after 100 cycles. No failure was observed after these 100 cycles.

**Figure 7:**
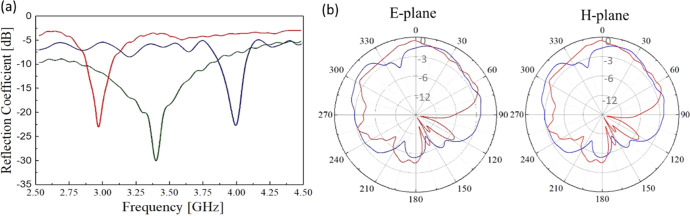
Solid textile antenna analysis. (a) Effect of meshes in the patch on solid ground and of both patch and ground on the change of resonant frequency compared to conventional equivalent. S11 of conventional textile antenna, meshed patch over the solid ground, and meshed patch over meshed ground plane are presented with blue, red, and green lines, respectively. (b) Measured and simulated radiation patterns in E and H planes at 4 GHz. Red lines represent simulated where the blue line is obtained from the measurement.

To characterize the resonance frequency of the antennas, the samples were connected to the VNA by using a lightweight coaxial cable. Prior to the measurements, a short-open-load (SOL) calibration was performed by using a Keysight 85052D calibration kit. Figure 7a shows that the fabricated solid textile microstrip antenna resonates at 4 GHz, whereas the antenna with the meshed patch over a solid ground plane and the fully meshed microstrip antenna has a resonance at 2.98 and 3.45 GHz, respectively. In conventional microstrip antennas, the resonant frequency is a function of some physical parameters such as the patch length and the substrate thickness. To shift down the resonant frequency, the patch length would need to increase.^50^ However, the obtained results suggest that meshing the antenna, either the patch or the ground plane, can also be used to tune the resonant frequency to the desired frequency spectrum. In our case, instead of increasing the length of the patch to lower down the resonant frequency, the meshed patch with a solid ground plane reduced the resonant frequency by 25% while the fully meshed antenna moved it down by 13.7%. The radiation patterns in the E-plane and H-plane for the solid textile antenna resonating at 4 GHz (Figure 7b) represent a relatively large back lobe, probably due to the small thickness of the silver layer in the textile ground plane.

**Figure 8:**
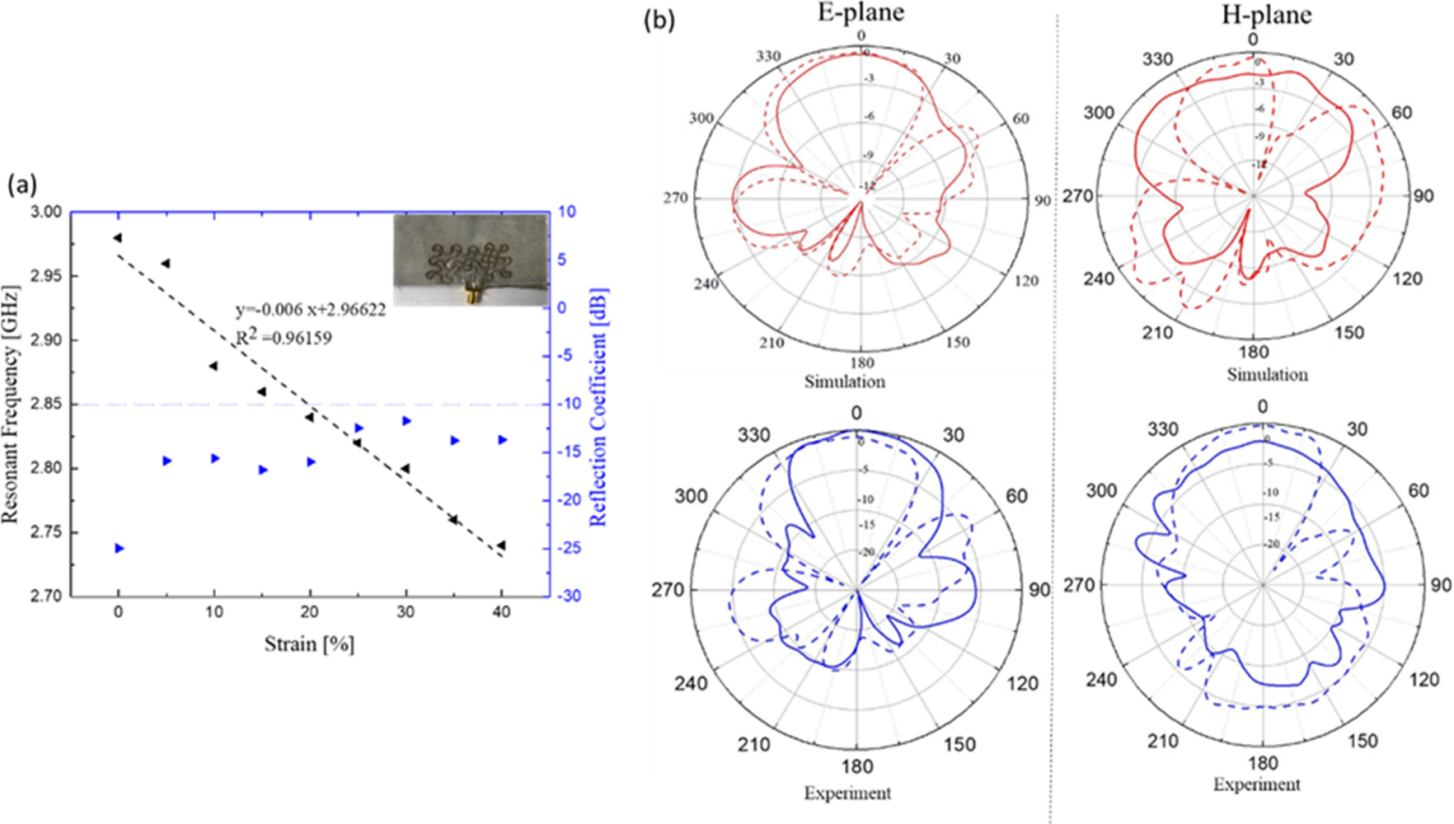
Textile meshed patch over solid ground analysis. (a) Change of resonant frequency and S_11_ as a function of tensile strain up to 40%. (b) Comparison of the radiation patterns in the E-plane (left) and H-plane (right) in relaxed (*f*_res_ = 2.98 GHz) and 30% uniaxial strain (*f*_res_ = 2.81 GHz) conditions. The simulation results of 30% strain (bottom) were extracted from the model obtained by the DIC method at 30%. Solid lines and dashed lines represent 0% and 30% strain, respectively.

The textile meshed patch over the solid ground plane showed good stretchability up to 40%, with the resonant frequency shifting down with a linear trend as shown in Figure 8a. It was observed that the resonant frequency moved down to 2.75 GHz when the uniaxial strain increased up to 40%. The reflection coefficient values were kept below −10 dB within the entire elongation range, which indicates that the impedance matching between the antenna and the inset feedline was maintained over the stretching. The linear trend suggests that the antenna has the potential to be used as a strain sensor by exploiting its stretchability, as previously stated. Considering the sensitivity to strain as , the average sensitivity of the antenna is 0.2 over the 0–40% strain range. To study the effect of strain on the radiation pattern, the antenna was placed on the tensile setup with an applied strain of 30%. The radiation patterns in the unstretched and stretched conditions were then compared. As observed from Figure 8, the radiation pattern in the E-plane did not change significantly when the antenna was stretched, but in the case of the H-plane, a null appeared in the main lobe. In general, the gain of the antenna increased when the strain was applied, which is in line with previous work,^45^ which proved that the losses in the meshed transmission line decreased when the device was stretched.

**Figure 9:**
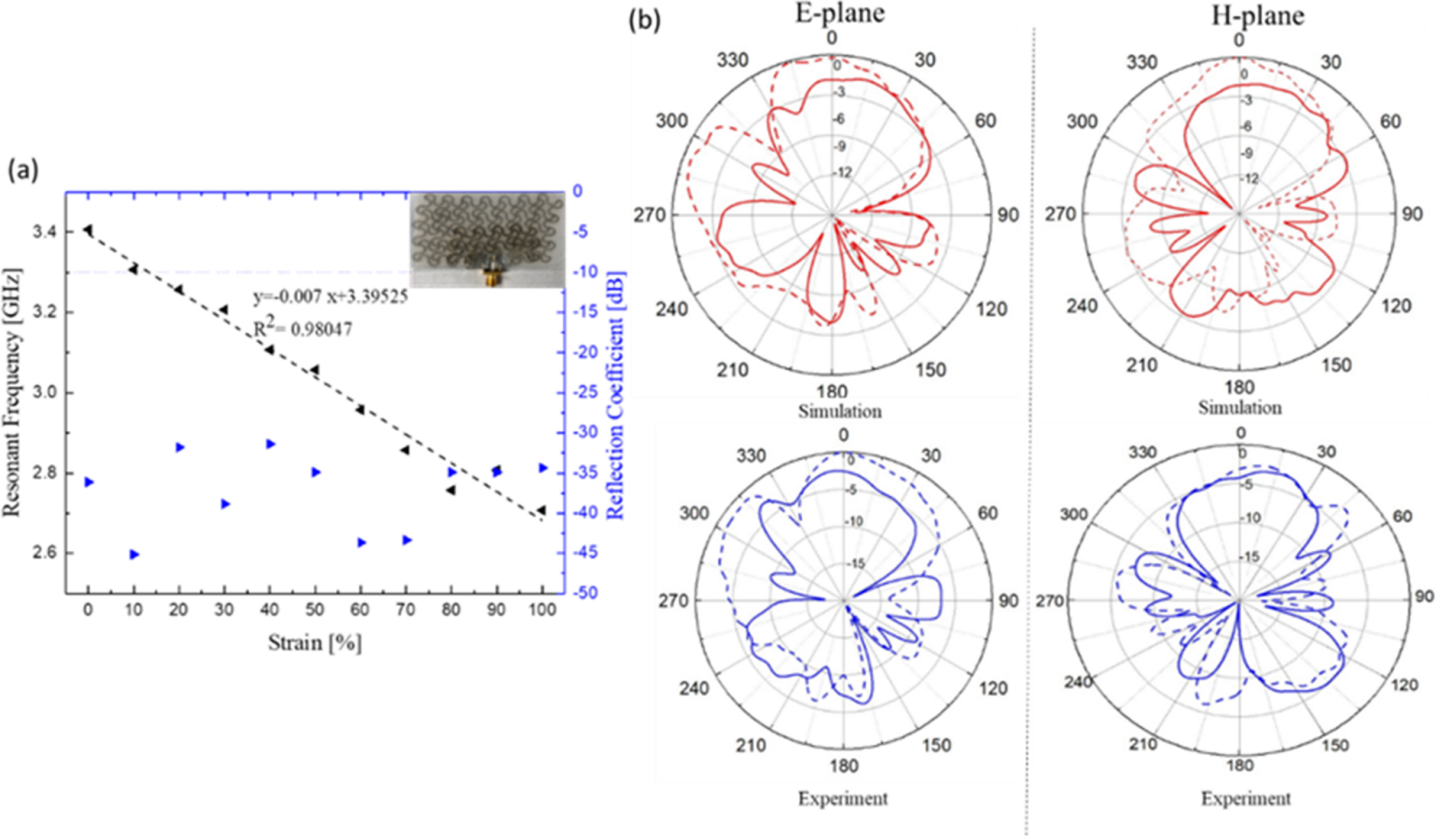
Textile meshed patch over meshed ground analysis. (a) Change of resonant frequency and *S*_11_ as a function of tensile strain up to 100%. (b) Comparison of the radiation pattern in E-plane (left) and H-plane (right) in relax (*f*_res_ = 3.45 GHz) and 30% uniaxial strain (*f*_res_ = 2.75 GHz). The simulation results of 30% strain (bottom) were extracted from the model obtained by the DIC method at 30%. Solid lines and dashed lines represent 0% and 30% strain, respectively.

The EM of a fully meshed microstrip antenna (i.e., both patch and ground planes patterned by using serpentine mesh) as a function of the tensile strain was also characterized. Figure 9a shows that the resonant frequency shifted downward linearly from 3.45 to 2.75 GHz when the antenna was stretched from 0% up to 100% of uniaxial strain. These results indicate that the antenna could be used to measure large strain wirelessly (up to 100%) with an average sensitivity of 0.25. While the inset feed line was designed to match the impedance for the solid microstrip antenna, it was observed that the same design implemented for the meshed versions maintained good impedance matching even over the 100% uniaxial strain (Figure 9a). The radiation patterns in the E-plane and H-plane are shown at 3.45 GHz (0% strain) and 3.2 GHz (30% strain) in Figure 9b. It can be observed that the half-power beam width of the proposed antenna is very wide, whereas the notable back lobe specifies that some power is lost as backradiation.

**Figure 10:**
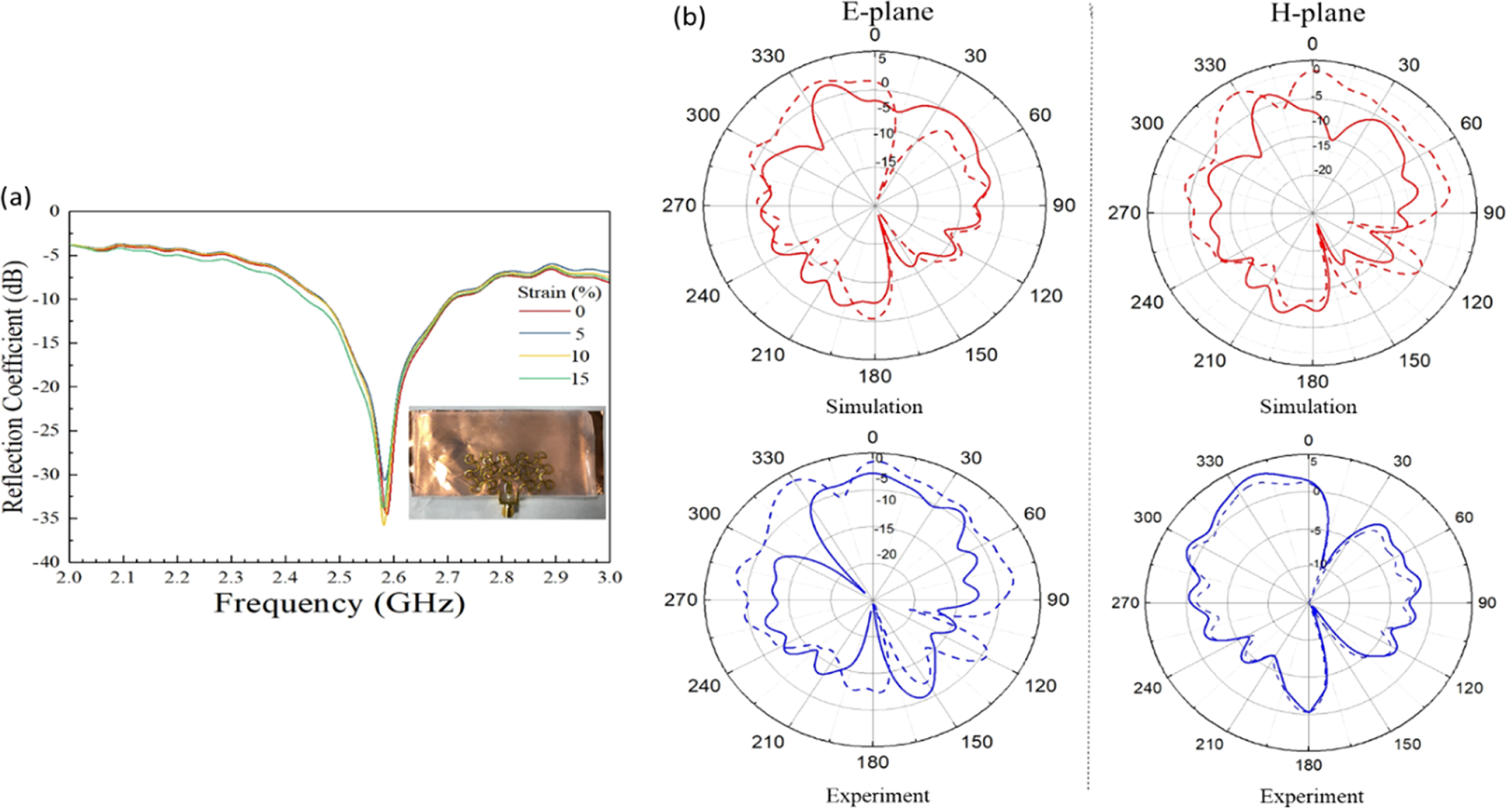
Gold meshed patch over a metallic solid ground analysis. (a) Measurement of *S*_11_ as a function of frequency as strain increase up to 15%. (b) Comparison of the radiation pattern in E-plane (left) and H-plane (right) in relax (*f*_res_ = 2.58 GHz) and 15% uniaxial strain (*f*_res_ = 2.75 GHz). The simulation results of 15% strain (model obtained by the DIC method at 15%) are compatible with the experimental results. Solid lines and dashed lines represent 0% and 15% strain, respectively.

When comparing to the fabricated gold meshed-patch antenna, Figure 10a shows that the resonance frequency (2.58 GHz) of gold meshed patch is lower than the textile-based counterpart antenna even if they have the same dimension (*f*_res_ = 2.98 GHz). This is because, owing to the structure of the textile, the actual electrical length of the patch in textile-based antenna is longer than in the gold antenna. The silver-plated knitted textile contains a series of interlocking loops so the actual electrical length is longer than the physical length. In addition, it was observed that applying uniaxial strain on the gold meshed-patch antenna did not have any notable effect on the resonant frequency as it happened for the textile version (see Figure 10a).

In addition, it was observed that applying uniaxial strain on the gold meshed-patch antenna did not have any notable effect on the resonant frequency as it happened for the textile version (see Figure 10a). Moreover, it was the sample was examined for stretchability, and it could not withstand strains beyond 20%, the point at which cracks started to appear and the signal was lost.

The radiation patterns in the E-plane and H-plane (Figure 10b) show notable differences between this pattern and its textile-based counterpart. This could be explained due to the differences in the surface morphology between the silver-coated textile and gold, which cause a notable discrepancy in the surface current density on the patches. Indeed, it can be said that the knitted structure of the textile caused surface current phase cancellations in the loop yarn, thus not contributing to the far-field radiation.

Considering the properties of the developed antenna in terms of flexibility, stretchability, wearability, and conformability, the device can be used as a mechanical strain sensor on curvilinear surfaces such as the human skin to monitor the movement of the human body. To this end, the fully meshed microstrip antenna was placed on the elbow for movement detection. Defining the bending angle (θ) as the angle between the arm and the forearm, the motion was captured by the decrease in the resonance frequency of the antenna (as shown in Figure 12a).

**Figure 11:**
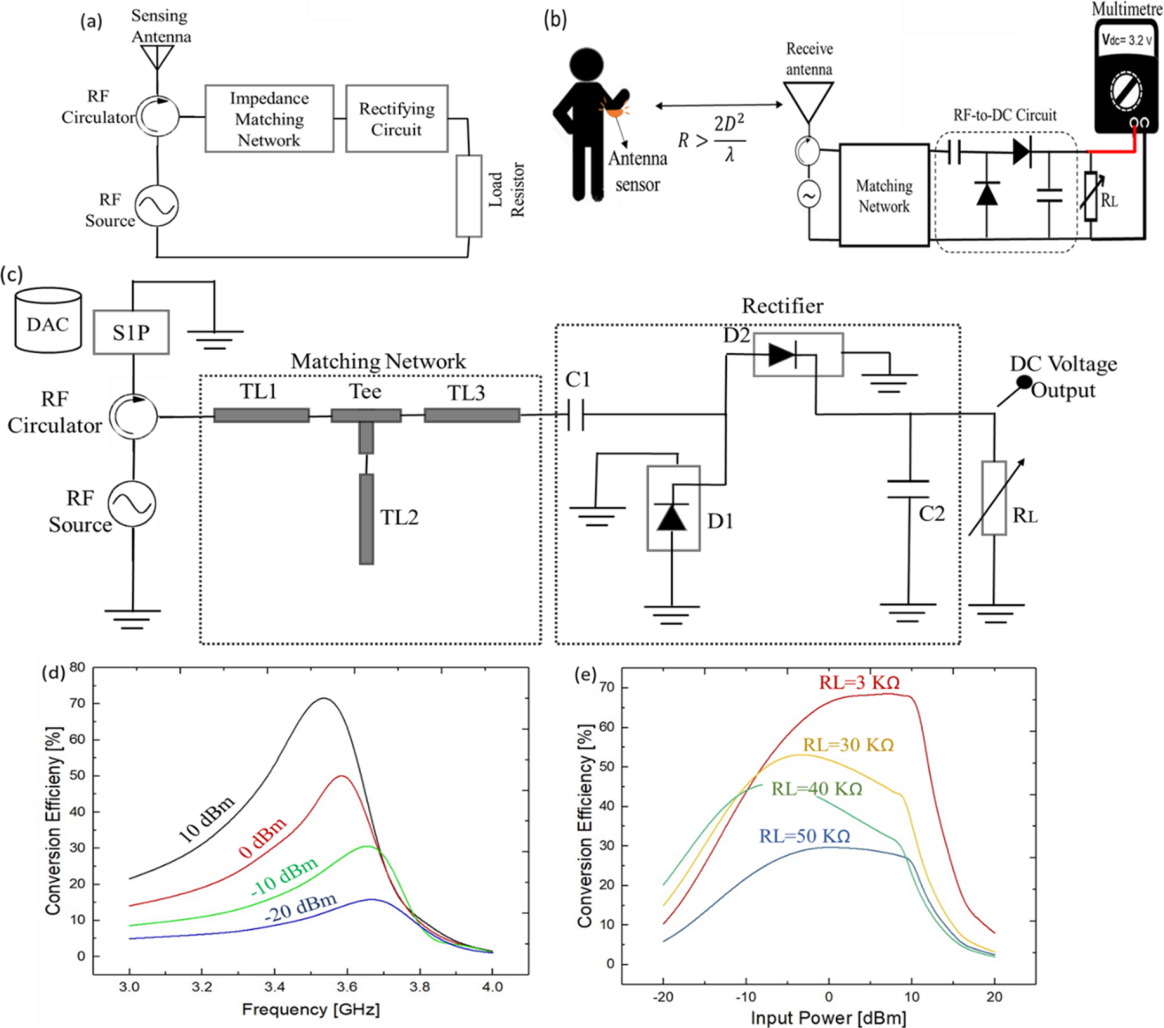
(a) Block diagram of the proposed readout circuit to convert frequency shift of stretchable sensing antenna to DC voltage. (b) Measurement setup to translate the bending angle of human arm to DC voltage wirelessly with the stretchable antenna. (c) Schematic of the rectifying circuit with a single stage RF rectifier simulated in ADS. (d) Conversion efficiency at different input power levels of −20, −10, 0, and 10 dBm, where the load resistor is 30 kΩ. (e) Conversion efficiency at different input powers for some values of the load resistor.

A linear trend was observed when measuring the resonant frequency of the antenna for different bending angles ranging from 120° to 40°. This way of detecting the bending angle is also attractive for applications such as robotics and prosthetics, where wireless strain sensors can provide an accurate measure of joint angles, without adding to the traditional issue of wiring complexity. In addition, it is possible to use the antenna as a mechanical strain sensor to remotely control the robotic movements in a manner.^51^ In such areas, the fully sensing antenna can provide feedback and trigger the actuator by converting the resonance frequency variation into a variation in DC voltage. This can be achieved by using an RF to DC rectifier.^52^ Once the information is processed (i.e., the change in the resonance frequency can be acquired and translated to a particular bending angle), it can be transferred to a robotic hand controller for movement control. Figure 11a illustrates the block diagram of the readout RF circuit for the proposed sensing antenna. The output DC voltage can be used as an actuating signal.

The antenna in Figure 1 has been realized to transduce the stretching into a variation in the resonance frequency. To characterize the behavior of the realized antenna under different stretching conditions, the VNA has been used to measure the *S*_11_ parameter and the amount of shift due to the stretch. The results of this characterization are used to create a calibration curve as shown in Figure 9. However, for the implementation in real applications, an RF readout circuit has been proposed which converts the reflected power from the proposed antenna sensor into a DC voltage by rectification. The VNA characterization helped as well to identify the frequency range shift to design the RF readout circuit and to help select the appropriate RF diode. Therefore, for potential applications the RF readout circuit is the one which is going to be used. The DC output represents the amount of stretch applied to the antenna and can be used to actuate other devices.

The reading principle to detect the deformation in a stretchable antenna is based on the measurement of reflected power from the sensing antenna. A detection circuit for the stretchable sensor antenna can be apprehended with an RF source that emits an RF signal at the resonance frequency of the antenna. The received reflected power can be directed by using a three-port circulator where the reflected power can be converted into a DC voltage by using an RF rectifying circuit. Good impedance matching at the third port of the circulator ensures that the maximum power is transferred to the rectifying circuit. The proposed setup in Figure 11b enables to map the bending angle to DC voltage variation. The receiving antenna can be a microstrip patch antenna working at 3–4 GHz.

An Advanced Design System (ADS, Keysight Technologies, Santa Clara, CA) was used to simulate the rectifier system along with the impedance matching network. Considering the operating frequency range of the antenna, FR4 with a thickness of 20 mil is set as a substrate. The *S*-parameter touchstones files (at different bending angles), collected from the VNA in the previous experiment, were assigned to the schematic by using the Data Access Component (DAC). The DAC component enables to sweep of multiple imported data files. A three-port RF circulator is a passive device designed by a Y junction symmetrical stripline coupled to a magnetic biased ferrite material. Once the RF signal generated by using an RF source and the second port are well matched (i.e., operating at the resonant frequency), the majority of the emitted power (consider an ideal circulator with negligible insertion loss) will be absorbed by the antenna. In this case, the reflected signal to the third port will be minimum. On the other hand, if the impedance is mismatched at the second point, in our case due to deformation of the antenna, the reflected power directed toward the third port will be greater. Good impedance matching at port three is key to direct maximum power to the rectifier circuit.

The next step is to design L-network matching and a full-wave single-stage rectifier. The optimum lengths of the microstrip lines were calculated by using the LineCalc tool. The widths of microstrip lines are 3 mm, compatible with conventional PCB fabrication processes. A T-junction between the transmission lines is considered with lines width match to the adjacent transmission lines to avoid parasitic junction effects. A T-junction between the transmission lines is considered with lines width match to the adjacent transmission lines to avoid parasitic junction effects.

In addition, a full-wave rectifying circuit was designed by using two HSMS-2850 diodes in a series configuration. HSMS-2850 is a Schottky detector diode with zero DC bias and high detection sensitivity and a low parasitic capacitance of 0.08 pF, suitable to use from 9.5 MHz to 5.8 GHz according to the manufacturer datasheet. The diode D1 is functional at the positive cycle where the diode D2 is working at the negative cycle, storing energy in capacitors C1 and C2. A SOT-23 package with typical package inductance and capacitance of 2 nH and 0.08 pF is selected to be mount on the substrate. A parasitic series resistance of 10 Ω is chosen to modify the default characteristic of the diodes. The optimum values of distributed elements and capacitors are specified in Table 2. Because the diodes are nonlinear devices at microwave frequencies, the harmonic-balance (HB) method is performed to analyze the circuit. HB is a frequency domain simulator in which the linear devices of a circuit are modeled in the frequency domain where the nonlinear devices are modeled in the time domain and before each iteration is Fourier transformed. The rectifier circuit along with network matching system design in the ADS is depicted in Figure 11c.

**Table 2:** Optimal Parameters of the Discrete and Lumped Elements Obtained by Using the LineCalc, Optimization, and Tuning Tools in ADS

discrete elements	length (mm)	width (mm)	lumped elements	value
TL1	12.286	3	C1	30 pF
TL2	9.8	3	C2	300 pF
TL3	8	3	*R*_L_	1–100 kΩ

The conversion efficiency of the RF to DC is defined from eq 1
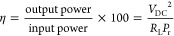
1in which *P*_r_ is the reflected power received in the rectifying circuit. It is noted that the conversion efficiency is a function of the load resistor and the received power. Figure 11d shows the conversion efficiency at different input power levels of −20, −10, 0, and 10 dBm. The conversion efficiency is increasing along with an increase in RF input power. At 0 dBm the efficiency is below 50% where the efficiency is at the highest obtained value of 71% at 10 dBm input power. Moreover, to achieve the peak output DC voltage, a load resistor is varied in the range 1–100 kΩ to study the effect of load resistance on the output voltage and the efficiency. It was noted that for 75K the DC voltage is 4.6 V at 10 dBm; however, the efficiency is as low as 12%. The conversion efficiency versus input power for some values of load resistor is depicted in Figure 11e. To find a trade-off between the output DC voltage and the efficiency, a load resistor of 30 kΩ has been chosen for the rest of the analysis.

**Figure 12:**
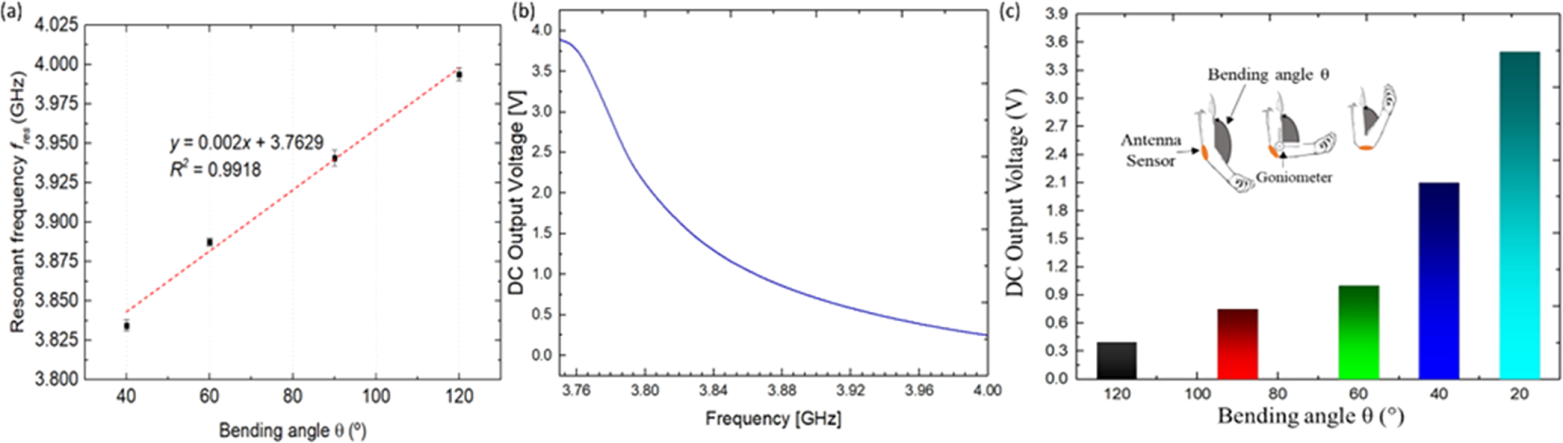
(a) Change in the resonant frequency of the antenna attached to the elbow for different bending angles. The variation in the resonant frequency follows a linear trend for the tested bending angles. (b) Simulated output DC voltage at an input power of 0 dBm. (c) Variation of produced DC voltage associated with the change of bending angles.

Figures 12b,c show the output DC voltage variation of the proposed design for load resistance of 30 kΩ where the input power is set at 0 dBm. The simulated maximum value of *V*_DC_ = 3.5 V is realized when the input data of the sensing antenna are associated with 20° bending of a human hand. The graph shows that in a rest position the DC voltage is minimum; however, the voltage is increasing when the hand is folded. Thus, reported work can lead to remote actuation of a robotic arm to mimic the human hand movement in real time (as shown in Figure 1 for potential applications). It means the performed movement by a person who is wearing the stretchable antenna could be reflected by a robot.

## V Conclusions

In this work, three versions of textile-based microstrip antennas have been designed, fabricated, and characterized. The antenna patch has a mesh structure over meshed and unmeshed ground planes using conductive textile. The EM characteristics of the meshed patch antenna were compared with its metallic counterpart fabricated with lithography. The meshed patch over solid/meshed ground plane demonstrated high stretchability (up to 40% and 100%, respectively) upon the uniaxial tensile strain. Both antennas presented a linear trend of their resonant frequency shift in response to increasing elongation, with experimental sensitivities of 0.2 and 0.25, respectively. Radiation patterns were also characterized upon the uniaxial tensile strain. The 2D DIC technique was implemented as a model for the deformed antennas to validate experimental results with FE simulation. As a proof-of-concept, the fully meshed antenna is shown to predict the joint angle of a human hand, which could potentially be extended to remotely control the robotic hand. Once the information is processed (i.e., the change in the resonance frequency is acquired and translated to a particular bending angle), it can be transferred to a robotic hand controller for the movement of a robotic arm. A rectifying circuit including two Schottky diodes (HSMS-2850) and an L-matching network is simulated in ADS to convert the RF signal to DC voltage. The proposed design obtained a conversion efficiency of 71% at 10 dBm input power over the load resistor of 3 kΩ. The obtained results indicate that stretchable antennas can be designed and fabricated by using conductive textile with several advantages such as low cost, easy integration into fashion garments, and self-strain sensing capabilities. In this way, the number of components attached to the garments can be reduced since the transmitting antenna is also used as a strain sensor.

## Supporting Information Available

Electromechanical characterization (Figure S1) (PDF)
