# Supplementary material for: Textile-Based Stretchable Microstrip Antenna with Intrinsic Strain Sensing
Source: ACS Appl Electron Mater. 2021 May 3;3(5):2233–46. doi: 10.1021/acsaelm.1c00179 (PMC8289303; doi:10.1021/acsaelm.1c00179)
Supplement: Supplementary file 1 — el1c00179_si_001.pdf [file el1c00179_si_001.pdf]

Supporting Information

Textile based stretchable microstrip antenna with intrinsic strain sensing

*Fatemeh Nikbakhtnasrabadi, Hatem El Matbouly, Markellos Ntagios, Ravinder Dahiya**

Bendable Electronics and Sensing Technologies (BEST) Group,

University of Glasgow, G12 8QQ, UK.

Corresponding Author E-mail: Ravinder.Dahiya@glasgow.ac.uk

The custom-made tensile setup shown in **Figure S1** was used to study the change in the electrical properties of the textile (i.e. resistance) upon uniaxial stretching. The setup consists of two holders controlled by a pair of stepper motors (Translation Stage VT-21L Micronix, US) with a velocity of movement of $V_S = 0.1$ mm/s and a LabVIEW to stretch the samples homogenously from both sides up to 100% strain. Two rectangular textile pieces were added to the design of the patch (shown as black bars in **Figure 6** inset) to make electrical connections. Electrical resistance was simultaneously collected using a digital multimetre (34461A, Agilent, US) connected to the samples.

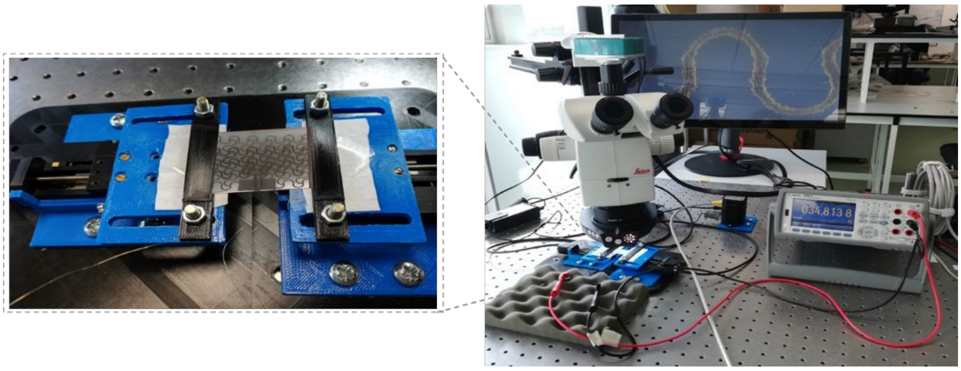

Figure S1. Custom-made setup to evaluate the electromechanical performance of meshed textile structure.
